# Effect of Low-Level Laser Therapy on Early Wound Healing and Levels of Inflammatory Mediators in Gingival Crevicular Fluid Following Open Flap Debridement

**DOI:** 10.7759/cureus.34755

**Published:** 2023-02-07

**Authors:** Priyanka Misra, Rupali Kalsi, Sachit Anand Arora, Kumar Saurav Singh, Simoona Athar, Anchal Saini

**Affiliations:** 1 Department of Periodontics, ITS Dental College, Greater Noida, IND; 2 Department of Periodontology, Government Institute of Medical Sciences, Noida, IND

**Keywords:** pocket therapy, periodontitis, open flap debridement, gingival crevicular fluid, low level laser therapy

## Abstract

Introduction

Low-level laser therapy (LLLT) has a beneficial effect on pain relief and wound healing. This study aims at a clinical evaluation of early wound healing and a biochemical evaluation of inflammatory mediators in gingival crevicular fluid (GCF) following LLLT with an open flap debridement (OFD) in periodontal therapy.

Material and methods

This randomized controlled trial included 40 chronic periodontitis patients with bilateral attachment loss, pocket depths of 5 mm affecting at least two quadrants, and radiographic evidence of horizontal bone loss. 120 control sites were randomly selected to receive OFD, and contralateral 120 test sites received bio-stimulation with a diode laser (890 nm) after OFD. The wound healing index was recorded at the 1st and 2nd weeks, and clinical parameters such as the plaque index, gingival index, pocket probing depth, clinical attachment level, and GCF inflammatory mediators were evaluated at baseline, 3, and 6 months.

Results

From the start of the study to 6 months later, there was a statistically significant drop in plaque index, gingival index, probing pocket depth, and gain clinical attachment levels in both groups. However, when the two groups were compared, there were no significant differences at any time intervals. GCF inflammatory mediators tumor necrosis factor (TNF) alpha and matrix metalloproteinases (MMP-8) decrease, and osteoprotegerin (OPG) levels increase in both the test group and control group from baseline to 3 months and 6 months. In intergroup comparisons, there was a statistically significant reduction in the test group as compared to the control group at 6 months. There was a decline in gingival crevicular fluid - interleukin-6 (GCF IL-6) levels from baseline to 3 months and 6 months in both the groups but when analysed statistically, the results were not significant on intergroup and intragroup comparison at any time interval. The Landry Wound Healing Index values in the 1st and 2nd weeks were showing statistically significant improved healing in the test group as compared to the control group. There was significantly better wound healing at sites where a diode laser was used.

Conclusion

LLLT increases early wound healing after periodontal surgical procedures.

## Introduction

Periodontitis is an infectious bacterial disease characterized by loss of attachment and bone supporting the sockets. If left unattended, it leads to gingival recession and pocket formation, which eventually results in early exfoliation of the teeth. It is primarily caused by pathogenic plaque microflora and is altered by systemic factors, resulting in direct or host-mediated tissue injury. The basic motive of periodontal therapy is to halt the disease's progression by removing and altering the factors causing the disease by either surgical or non-surgical means [1].

Lasers have become an indispensable part of periodontal therapy. Since the last decade, lasers have assisted successfully in almost every treatment procedure in periodontics. Maiman created the first laser apparatus in 1960, and Goldman used it in dentistry in 1964 [2]. Soft tissue surgical procedures using lasers were found to achieve hemostasis and sterilization with minimal post-operative pain in contrast to conventional surgical procedures. The conclusive goal of any periodontal therapy is to attain sustainable regeneration and simultaneously ensure that the healing period is brief and comfortable for the patient [2-4]. Low-level laser therapy (LLLT) has also been examined recently for its effectiveness in the medical and dentistry fields' wound-healing procedures. Low-level laser therapy, or photobiostimulation, was first proposed by Mester and his co-workers in 1967 [3].

LLLT refers to the non-invasive photon bombardment of tissues, which brings about multiple changes at the cellular and molecular levels. Adenosine triphosphate (ATP), ribonucleic acid (RNA), and protein synthesis are said to be increased as a result of the cytochrome c oxidase, a mitochondrial component of the cellular respiratory chain, absorbing high-energy photons. After an open flap debridement (OFD), lasers are better at regenerating periodontal tissue and reducing the number of germs in the mouth.

LLLT, or photobiological stimulation, brings about various biological changes that not only assist in the acceleration of healing wounds but also help in the attenuation of pain and postoperative discomfort. LLLT is also known as "soft laser therapy" and "laser biostimulation" [3]. The bio-stimulatory and inhibitory effects of LLLT are governed by the Arndt-Schulz law, wherein low doses increase physiological processes and strong stimuli inhibit physiological activity. LLLT with a diode laser has stimulatory effects on human gingival fibroblasts, which may have beneficial effects on healing wounds [4]. Wound healing certainly starts at the moment of injury, but the complete healing phase almost lasts up to 1 or 2 years. The tissue repair phase, which begins on the third day and lasts about two weeks, involves fibroblast proliferation and collagen genesis. The supplementation of LLLT at this point in time leads to expedited wound-healing events [4].

This study aims at a clinical evaluation of early wound healing and a biochemical evaluation of inflammatory mediators in gingival crevicular fluid (GCF) following LLLT with an open flap debridement (OFD) in periodontal therapy.

## Materials and methods

After getting approval from the institutional ethical committee (reference number IEC/Perio/4/19), 40 patients with Stage II Grade B periodontitis who were willing to participate in the study were recruited from the Department of Periodontology, ITS Dental College and Research Centre, Greater Noida, India as per case definition of American Academy of Periodontology (AAP) classification 2017 [5] with residual pocket depths of ≥ 5 mm and <7 mm post-phase 1 therapy affecting a minimum of three sites each in at least two quadrants and radiographic evidence of horizontal bone loss were included in the study. While patients with diabetes, hypertension, atherosclerosis, and other systemic diseases, including pregnant and lactating women, smokers (current or smoking within the last 5 years), patients allergic to medications, and patients with a recent history of antibiotic use (within the last 3 months) known to affect periodontal health, were excluded for the study.

The randomized controlled trial was carried out by tossing the coin where bio-stimulation with a diode laser (890 nm) after OFD was used to treat the site on one side (test group), while open flap debridement was used to treat the site on the other side (OFD; control group). The surgical site was anesthetized with a 2% lignocaine hydrochloride solution with adrenaline (1:80 000) in the control group (OFD). There were made intra-crevicular incisions. The roots were meticulously scaled and planed, and the granulation tissue was removed from the defects. There was no root surface conditioning carried out. After that, simple interrupted sutures were used to close the control sites. While in test groups, the patient and every member of the operating room staff were required to wear safety laser glasses. Sweeping movements were made with the tip in the pocket apico-coronally (vertically) and mesiodistally (horizontally). Laser bio-stimulation was carried out with a power output of 1.5 watts in non-contact mode at baseline, the third, and the seventh day after flap surgery for 30 seconds twice with a 60-second interval in the test group.

Each recall visit included a clinical evaluation of wound healing using the Landry index. According to redness, the presence of granulation tissues, bleeding, suppuration, and epithelialization, the healing index (HI) assigns a score to the healing process. A scale of 1 to 5 was used, with 1 representing very poor tissue repair and 5 representing good tissue healing. The healing is more effective the higher the score. According to the clinical assessment, this index rates the surgical wound. Landry wound healing index [6] was taken down at the 1st and 2nd weeks, and clinical parameters, viz., plaque index (PI), gingival index (GI), pocket probing depth (PPD), clinical attachment level (CAL), and GCF inflammatory mediators tumor necrosis factor (TNF) alpha, IL-6, matrix metalloproteinases (MMP-8), osteoprotegerin (OPG) were estimated at 0, 3, and 6 months. An enzyme-linked immunoassay (ELISA) kit (Chongqing Biospes Co. Ltd., Chongqing, China) was used to analyze the inflammatory mediators.

After gathering, sorting, and tabulating all the data, descriptive and analytical statistics were used. Since the data did not follow a normal distribution, a nonparametric test called the Shapiro-Wilk test was employed to examine the information. The Mann-Whitney U test and Wilcoxon signed-ranked test were employed to examine differences between the groups. Software called Statistical Package for the Social Sciences version 20.1 was used to conduct all of the statistical analyses (IBM Corp, Armonk, USA).

## Results

Two hundred and forty test and control sites from 40 systemically healthy periodontitis patients of both genders with probing pocket depths between 5 and 7 mm and radiographic evidence of horizontal bone loss were selected. Open flap debridement with and without application of LLLT was done in test and control groups, following which clinical parameters, GCF biomarkers, and the Landry early wound healing index were recorded at subsequent intervals.

Comparisons of clinical parameters (plaque index, gingival index, probing pocket depth, and clinical attachment levels) at baseline, 3 months, and 6 months are shown in Table 1.

**Table 1:** Comparison of the Clinical Parameters in the Test Group and Control Groups

Clinical Parameters	Time interval	Test Group N=120 sites	Control Group N=120 sites	Intergroup Comparison
		Mean ± SD	Mean ± SD	P value
Plaque Index	At Baseline	2.14± 0.39	2.32±0.32	0.056
	3 months	1.35±1.197	0.98±0.34	0.081
	6 months	0.83± 0.30	0.74±0.17	0.107
Gingival index	At Baseline	2.44± 0.24	2.06±0.48	<0.001
	3 months	1.25± 0.42	1.41±0.63	0.120
	6 months	0.98±0.41	0.91±0.35	0.443
Pocket Probing Depth	At Baseline	6.40± 0.64	6.47±0.54	0.549
	3 months	4.30± 0.87	4.09±0.74	0.272
	6 months	3.19±0.60	3.21±0.57	0.859
Clinical attachment level	At Baseline	3.22±0.65	3.26±0.90	0.756
	3 months	2.38±0.59	2.49±0.69	0.255
	6 months	1.42±0.35	1.54±0.72	0.220

From the start of the study to 6 months later, there was a statistically significant decrease in plaque index, gingival index, probing pocket depth, and clinical attachment levels in both groups. However, when the two groups were compared, there were no significant differences at any time.

GCF TNF alpha levels decreased in both the test group and control group from baseline to 3 months and 6 months. In intergroup comparison, there was a statistically significant reduction in the test group as compared to the control group at 6 months (p-value < 0.036). There was a decline in GCF IL-6 levels from baseline to 3 months and 6 months in both the groups but results when analyzed statistically were not significant on intergroup and intragroup comparison at any time interval (p-value < 0.265). GCF MMP 8 levels were decreased in both the test and control groups from baseline to 6 months. In intergroup comparison, the reduction in the test group was more than the control group and the results were statistically significant (p-value < 0.005). There was an increase in GCF OPG levels from baseline to 3 months and 6 months in both groups but the escalation in the test group was significantly higher than that of the control group (p-value < 0.023) (Table 2).

**Table 2:** Comparison of the Biomarkers in the Test Group and the Control Group TNF: tumour necrosis factor; IL: interleukin; OPG: osteoprotegrin; MMP: matrix metalloproteinase

Biomarkers		Test Group N=120 sites	Control Group N=120 sites	Intergroup Comparison
		Mean ± SD N=120 sites	Mean ± SD N=120 sites	P value
TNF alpha	At Baseline	27.34±6.39	28.10±6.03	0.556
	3 months	23.08±7.59	24.56±7.30	0.486
	6 months	20.39±4.73	21.47±4.22	0.036
IL-6	At Baseline	20.22±3.45	20.31±3.61	0.95
	3 months	17.51±2.8	18.78±3.3	0.37
	6 months	16.19±3.14	17.86±3.3	0.265
MMP 8	At Baseline	373.71±68.58	365.10±61.85	0.203
	3 months	211.18±53.46	240.80±59.85	0.139
	6 months	95.20±18.91	118.04±22.51	0.005
OPG	At Baseline	84.11±24.23	87.22±25.54	0.39
	3 months	173.30±30.01	158.70±28.30	0.138
	6 months	180.80±31.72	153.30±25.32	0.023

On comparison of the Landry Wound Healing Index, the baseline values for the test and control groups were 3±0.67 and 3.2±0.68, respectively, with no statistical difference in the scores. The values on the 7th day for the test and control groups were 3.5±0.67 and 2.5±0.50, respectively, showing statistically significant better wound healing in the test group (p-value < 0.001, S). The Landry Wound Healing Index values in the 2nd week were 4±0.49 and 3±0.75, showing again statistically significant improved healing in the test group as compared to the control group (p-value < 0.001) (Table 3).

**Table 3:** Intergroup Comparison of Wound Healing Using the Landry Wound Healing Index SD: standard deviation

	Test sites N=120	Control sites N=120	P-value of Intergroup Comparison
	Mean ± SD	Mean ± SD	
At Baseline	3±0.67	3.2±0.68	Not significant
1 week	3.5±0.67	2.5±0.50	<0.001, S
2 week	4±0.49	3±0.75	<0.001, S

## Discussion

Periodontitis is a prevalent disease all over the world. Environmental conditions, the immune response generated by the host, and pathogenic microflora all play an important role in the progression of the disease. Many recent studies have revealed that diode lasers have been successful in enhancing periodontal wound healing after surgical procedures. This study was done on 40 people with periodontitis to see how well LLLT helped early periodontal wounds heal after OFD and to compare clinical and biochemical parameters after OFD with and without laser bio-stimulation.

The PI reflects the patient's overall hygiene status; it is assessed postoperatively according to the index of Silness and Loe [7] which shows a trend toward progressively improving oral hygiene, whereas the GI records gingival inflammation. Dental plaque is a hub of microbes or biofilm that is embedded in an extracellular gelatinous matrix and contributes to periodontal disease [7]. The local cause of inflammation is the buildup of plaque, which causes frequent micro-ulcerations in the epithelium that lines the soft-tissue wall of a periodontal pocket [8]. To estimate the increasing soft tissue destruction, disease enhancement, and response to periodontal treatment, measurements of PPD and CAL are the most frequently used informative parameters [9]. In the present study, there was a statistically significant reduction in the scores of PI, GI, PPD, and CAL from baseline to 6 months in both groups, but the intergroup comparison failed to show a statistical difference. The explanation of improvement in both groups is possibly due to the efficiency done OFD along with proper oral hygiene practices followed by the participants on a regular basis in both groups.

Similar to our findings, Khan F et al. (2021) [10], Gokhale SR et al. (2012) [11], and Jonnalagadda BD et al. (2018) [12] did studies and reported non-significant results on the comparison of tests and control groups showing no additional benefits of diode laser over conventional flap surgery. Kolamala et al. (2022) [13], on the other hand, found that the clinical parameters of treatment with diode laser were better than treatment with OFD alone from the start to 6 months. Different lasers, including the diode, Nd:YAG, CO2, Er:YAG, and Er,Cr:YSGG, have been suggested and are anticipated to act as an alternative or supplemental therapy to traditional, mechanical periodontal therapy. Other benefits include hemostasis, reduced postoperative edema, a decrease in the number of microorganisms at the surgical site, fewer sutures required, quicker healing, and less postoperative discomfort. 

Periodontitis is a local inflammatory process, and invasion of the periodontium is mediated through inflammatory markers. It is observed that host monocytes, macrophages, fibroblasts, and endothelial cells respond immediately, secreting chemokines and inflammatory markers against bacteria and their toxins [7]. Due to the complexities of periodontitis, relying on a single biomarker may not yield relevant results. To assess biological changes, the GCF assays of four periodontal disease biomarkers (TNF, IL-6, MMP-8, and OPG) were estimated. A description depicting the source and clinical significance in states of health and disease is done, but the values reflect a wide variation in range, possibly due to methodological disparities in sampling, laboratory settings, storage temperature, and methods of assessment. But as compared to traditional methods of assessment, these biomarkers demonstrate optimum prediction of site-specific prognosis and outcome of therapy. Although precise cutoff values for these biomarkers cannot be determined, the increase and decrease of these biomarkers serve as an important tool for assessing biological events occurring at the molecular level in the pocket lining [4,14-18].

TNF-alpha primary sources are monocytes and macrophages and secondary sources are fibroblasts and endothelial cells. It induces the secretion of collagenase by fibroblast, resorption of cartilage and bone, and has been implicated in the destruction of periodontal tissues in periodontitis leading to the synthesis of IL-1 and prostaglandin E2 (PGE 2), also activating osteoclasts and thus inducing bone resorption. It has synergistic effects with the bone resorptive actions of IL-1b. When assessed, a statistically significant reduction was seen in TNF-alpha levels in the test group as compared to the control group. TNF-alpha plays a definite role in alveolar bone loss. This significant decline in TNF-alpha at 6 months in test sites indicates decreased osteoclastic activity and inflammation, which may lead to a conducive environment for bone regeneration. Literature suggests that prolonged LLLT applications are able to lower the tissue concentrations of TNF-alpha by almost 70% and help reach a level closer to healthy, non-inflamed gingiva [14]. Laser treatment reduces periodontopathic microbes, lowering TNF-alpha levels. This could be corroborated by a study performed by Aimbire F et al. (2006) [19], who found a reduction in TNF levels after low-level laser therapy in periodontal surgical procedures. The source of OPG is osteoblasts, which play a vital role in inhibiting the differentiation and activation of osteoclasts. A significant increase in GCF OPG levels is seen at 6 months in the test sites compared to the control sites. Bone regeneration is regulated by the interplay of different cytokines. OPG is an osseoprotective marker, and its increase depicts a reduction in the inflammatory process and signifies that the molecular mechanism of bone repair is upregulated in areas where LLLT was done. Huck et al. reported higher levels of salivary OPG in treated periodontitis patients in the maintenance phase than in untreated periodontitis patients [20].

MMP-8 primary sources are monocytes and macrophages, while secondary sources are the fibroblasts and endothelial cells. MMP-8 is a chief collagenase and depicts the state of health and disease in subgingival periodontal tissues. In our study, a decline in MMP-8 levels was seen in both test and control sites, signifying the transition from a state of disease to health following open flap debridement in both groups. There is a statistically significant decrease in the test group when compared to the control group, showing an additional benefit of LLLT on the healing apparatus. MMP-8 is described as a surrogate indicator of neutrophil density as it is stored in secretory granules and released during inflammation. MMP-8 and MMP-9 are the main collagenases for the degradation of type I and type III collagen [18]. Increased levels of host and microbial-derived proteinases (MMPs) are responsible for extracellular matrix degradation in periodontal disease. As a result, lower MMP-8 counts indicate a stable healing apparatus devoid of disease activity at LASER-treated sites compared to control sites.

Similar studies by Deshmukh et al. (2018) [21] and Saglam et al. (2014) [22] reported significant improvements in clinical parameters and lower MMP-8 levels in laser-treated periodontal disease sites. Sezen et al. (2020) [23], on the other hand, found no statistical difference in MMP-8 reduction between conventional flap surgery and laser-assisted surgery.

IL-6 levels' primary sources are the monocytes and macrophages, and secondary sources are fibroblasts and endothelial cells which are responsible for the production of acute-phase proteins. There was a decline in IL-6 levels from baseline to 6 months in both groups, but intergroup comparison failed to yield any significant changes, though a numerically greater reduction was observed in the sites with LLLT application. IL-6 is produced by different types of cells and expressed by both the host and bacteria. The decrease in IL-6 levels in both groups is indicative of healing events taking place in both groups, whereas insignificant differences in test and control sites are suggestive of the low precision potential of this biomarker in wound healing. Bolyarova et al. (2019) [24] discovered that combining SRP with LLLT reduces GCF IL-6 levels. Another study, by Kardeşler et al. (2011) [25], found that SRP alone resulted in a significant decrease in the IL-6 level concentration of GCF.

The diversity of the results in different studies strengthens the idea that the production of inflammatory mediators differs from site to site and from subject to subject. One possible reason for this difference is that the release of inflammatory mediators is affected by many things, such as genetics and different types of bacteria. Wound healing is a complex biological process that involves well-organized cellular and biochemical events. On comparison of Landry Wound Healing Index scores, there was a statistically significant improvement in wound healing at the 1st and 2nd week intervals in the test group as compared to the control group. The possible explanation for this could be the absorption of laser light by mitochondrial chromophores, causing photodissociation of nitrous oxide and enhanced enzymatic activity and ATP generation. Thus, LLLT expedites the activation of numerous intracellular signalling pathways, which bring about enhanced cellular proliferation, tissue repair, and regeneration [26]. The right amount of LLLT dose causes the growth of new endothelium and blood vessels, which promotes the creation of granulation tissue and speeds up the healing process. Increased revascularization rate, which is known to have a significant impact on the outcome of wound healing after periodontal surgery, is another effect of LLLT on wound healing. Lingamaneni S et al. (2019) [27] discovered that LLLT improves wound healing and keratinization after periodontal surgery. Various histopathological studies have proclaimed improved healing, collagen formation, and homogenization of gingival lamina propria following LLLT [28, 29]. In contrast to this, a study by Damante et al. (2004) [30] found no additional benefits of laser therapy. No postoperative problems or poor clinical recovery were associated with this treatment method, suggesting that using laser therapy for pocket therapy may not have any negative effects.

Limitations of the current study are that small sample sizes were used. Patients were monitored for a short duration and blinding was not done. The effect of LLLT was assessed on GCF biomarkers at 3- and 6-month intervals. Future studies with the evaluation of biomarkers at 7 and 14 days should be done to elicit their benefits on early wound healing. Although pain perception was not analyzed in our study, the inclusion of this parameter may have helped us access the pain-attenuating benefits of laser therapy. Our study adds to the evidence that using LLLT to help a wound heal faster is a good idea.

## Conclusions

When it came to the plaque index, gingival index, probing pocket depth, and clinical attachment levels, OFD showed similar results as that of laser treatment. The periodontal flap surgery wound healing was greatly improved by the use of a diode laser following OFD. Establishing efficient laser application techniques will enable the implementation of this innovative treatment in periodontology and increase patient comfort.
